# Mapping Species Distributions with MAXENT Using a Geographically Biased Sample of Presence Data: A Performance Assessment of Methods for Correcting Sampling Bias

**DOI:** 10.1371/journal.pone.0097122

**Published:** 2014-05-12

**Authors:** Yoan Fourcade, Jan O. Engler, Dennis Rödder, Jean Secondi

**Affiliations:** 1 LUNAM Université d'Angers, GECCO (Groupe écologie et conservation des vertébrés), Angers, France; 2 Department of Wildlife Ecology, University of Göttingen, Göttingen, Germany; 3 Zoological Research Museum Alexander Koenig, Bonn, Germany; Dauphin Island Sea Lab, United States of America

## Abstract

MAXENT is now a common species distribution modeling (SDM) tool used by conservation practitioners for predicting the distribution of a species from a set of records and environmental predictors. However, datasets of species occurrence used to train the model are often biased in the geographical space because of unequal sampling effort across the study area. This bias may be a source of strong inaccuracy in the resulting model and could lead to incorrect predictions. Although a number of sampling bias correction methods have been proposed, there is no consensual guideline to account for it. We compared here the performance of five methods of bias correction on three datasets of species occurrence: one “virtual” derived from a land cover map, and two actual datasets for a turtle (*Chrysemys picta*) and a salamander (*Plethodon cylindraceus*). We subjected these datasets to four types of sampling biases corresponding to potential types of empirical biases. We applied five correction methods to the biased samples and compared the outputs of distribution models to unbiased datasets to assess the overall correction performance of each method. The results revealed that the ability of methods to correct the initial sampling bias varied greatly depending on bias type, bias intensity and species. However, the simple systematic sampling of records consistently ranked among the best performing across the range of conditions tested, whereas other methods performed more poorly in most cases. The strong effect of initial conditions on correction performance highlights the need for further research to develop a step-by-step guideline to account for sampling bias. However, this method seems to be the most efficient in correcting sampling bias and should be advised in most cases.

## Introduction

A key issue in ecology and conservation biology is to determine how species are distributed in space. Since extinction risk is associated with range size [1], a significant reduction of a species range often determines change in conservation status (see for example IUCN criteria [2], [3]) and prime conservations actions [4], [5]. Likewise, protected areas usually focus on biodiversity hotspots [6] in order to conserve efficiently as many species as possible [7]–[9]. Therefore, conservationists often need precise assessments of species ranges. Beyond simple range description, identifying which main factors limit distributions is essential to efficiently forecast the benefits of conservation management. In order to deal with these questions, several methods of species distribution modeling (SDM), also known as ecological niche modeling (ENM) [10], have been developed since the 1980s [11].

The principle of SDM is to relate known locations of a species with the environmental characteristics of these locations in order to estimate the response function and contribution of environmental variables [12], and predict the potential geographical range of a species [13]. These models estimate the fundamental ecological niche in the environmental space (*i.e.* species response to abiotic environmental factors [14]) and project it onto the geographical space to derive the probability of presence for any given area or, depending on the method, the likelihood that specific environmental conditions are suitable for the target species [15]. Distribution models are used by conservation practitioners to estimate the most suitable areas for a species and infer probability of presence in regions where no systematic surveys are available [16]. They can also assess the potential expansion of introduced species in newly colonized areas [17], [18], estimate the future range of a species under climate change [18], [19] or assist in reserve planning [20].

Several statistical models exist to predict the distribution of a species [21]. Beyond classical regression methods (Resource Selection Function RSF [22], [23], Generalized Linear Models GLM [24]), algorithmic modeling based on machine learning (for example Artificial Neural Networks [25], Maximum Entropy MAXENT [26], Classification And Regression Trees CART [27]) have become increasingly popular in recent years. Among these, MAXENT has been described as especially efficient to handle complex interactions between response and predictor variables [15], [28], and to be little sensitive to small sample sizes [29]. This, as well as its extreme simplicity of use, has made MAXENT the most widely used SDM algorithm. In December 2013, 1886 citations of the article describing the method [30] were reported in Web of Science.

MAXENT modeling, and SDM in general, is now commonly implemented in conservation-oriented studies [31]. Regional or continent-wide studies are facilitated by the recent availability of global datasets. Environmental layers, such as the global climate variables developed in the WorldClim project [32], offer continuous description of very large areas [33]. Similarly, the development of open biodiversity databases (see for example the Global Biodiversity Information Facility, GBIF, http://www.gbif.org) increases manifolds the spatial coverage of fieldwork observations that could have been collected by a single project. Such databases usually provide presence-only data that can be handled by modeling methods like MAXENT.

However, datasets derived from opportunistic observations or museum records rather than from planned surveys often exhibit a strong geographic bias [34], some areas being visited more often than others because of their accessibility [35] or their naturalistic interest. This unequal survey coverage of a species distribution is often referred as sampling bias, sample selection bias or survey bias. The quality of the model can be strongly affected if entire parts of the environmental space suitable to a species are absent or poorly represented in the survey dataset [36], [37], or alternatively, if some areas are overrepresented due to locally high sampling efforts. Several studies questioned the effect of sampling design [38], or the biased nature of museum and herbarium datasets [39] on the predictive performance of SDMs. Surprisingly, the issue of quantifying and correcting sampling bias has been poorly addressed despite its crucial importance. Although authors pointed out that the distribution of locations in the geographical and/or ecological space may impact the reliability of the model [35], [36], [40]–[43], the potential effect of the sampling bias in the dataset is usually poorly taken into account or not considered at all. However, very different SDM outputs can be generated that lead to contrasting conclusions whether sampling bias is corrected or not [44], making SDM studies that did not incorporate this issue highly doubtful.

In regard to the considerable influence of sampling bias on SDM prediction ability, Araújo et al. [45] considered the improvement of sampling designs as one of the five major challenges for future development of SDMs. Several bias correcting methods have been proposed [46]–[51] but they have been rarely used so far. Comparison and evaluation of different methods to correct sampling bias have only been recently carried out and no consensual guideline emerged to solve it. A few recent studies explored the consequences of and potential solutions to correct for sampling bias (by Syfert et al. [41], Kramer-Schadt [52], Varela et al. [53] and Boria et al. [54]). In spite of their interest, authors investigated a single case study so that it is not possible to evaluate the efficiency of a correction method across species. Second, the empirical bias caused by sampling intensity [41] was never tested and no more than two correction techniques have been evaluated at the same time whereas many more have been proposed or used in the literature [47], [49], [55]. Therefore, the influence of the nature and intensity of bias on the capacity of various techniques to correct for sampling bias has not been investigated. This remains however a critical issue, especially for users who need robust and reliable SDM predictions such as conservation practitioners.

**Figure 1:**
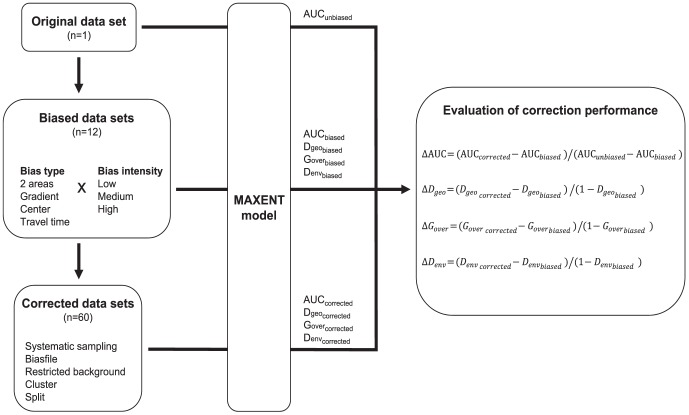
Workflow used in analyses. Original datasets of a virtual and 2 real species were altered to create 12 bias combining 4 bias types and 3 bias intensities. Five methods of sampling bias correction were employed to assess the improvement in the modeled distribution relative to the original distribution using MAXENT. Correction performance was assessed using AUC and 3 measures of overlap between the corrected the original unbiased model.

The goal of this comparative study is to test the effect of bias type, bias intensity, and correction method on MAXENT model performance. Unlike the previous cited studies [41], [52]–[54] we assessed the performance of five bias correcting methods among the most frequently used under various conditions of bias type and intensity. We used a virtual species to generate four types of sampling biases and three bias intensities, and applied on these biased datasets different corrections. We quantified the relative correction performance across the range of bias conditions and across species. The same framework was also applied on two real datasets. The full workflow on which analyses were based is sketched in Figure 1. Therefore, the present study provides for the first time a comprehensive multi-species evaluation of the most common methods of sampling bias correction under different scenarios of bias and intensities of bias. Intended for conservationists who use MAXENT on a regular basis, we expect this work to provide insights on the selection of the most suitable methods to produce reliable distribution models using biased datasets. Furthermore, it encourages modelers to develop improvements of techniques to correct for sampling bias suitable for the vast set of modeling methods available.

## Materials and Methods

### Species datasets

**Figure 2:**
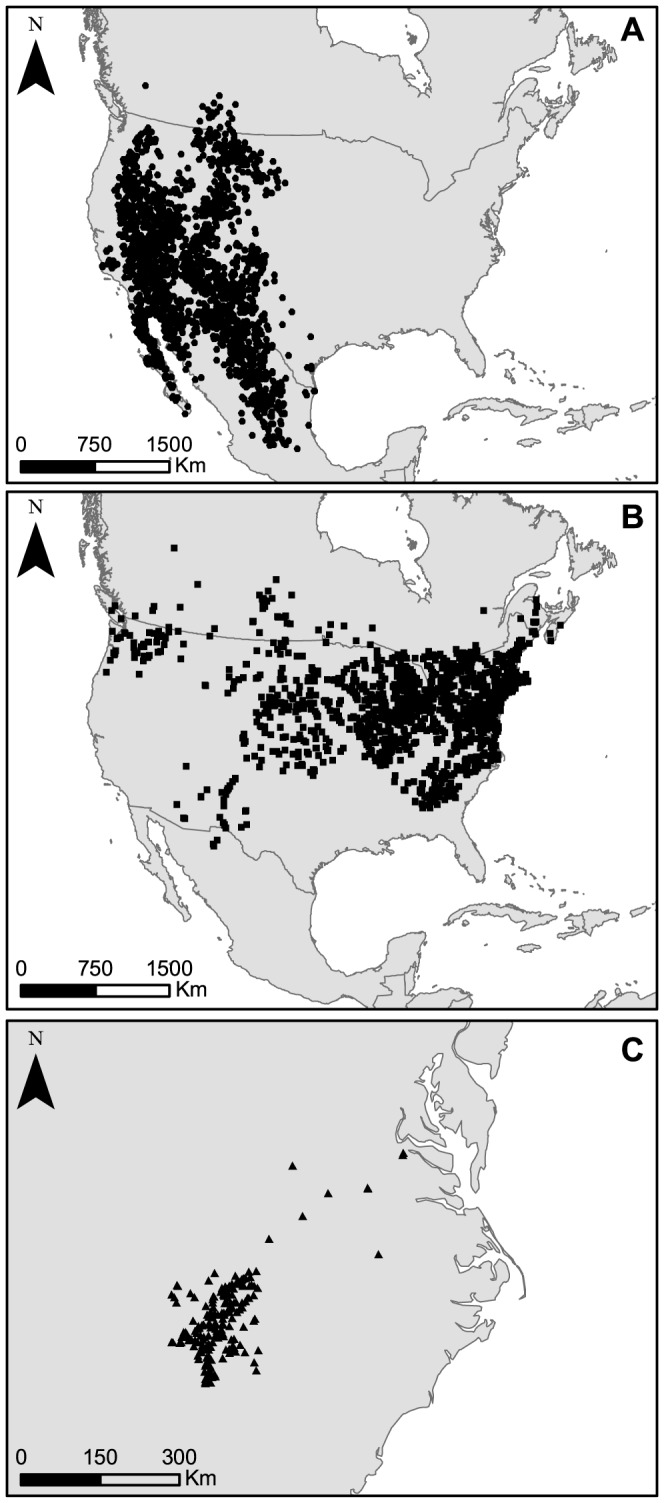
Locations of records used for modeling. (A) Virtual species; (B) *Chrysemys picta*; (C) *Plethodon cylindraceus*

In order to obtain a true unbiased dataset, we created a virtual species by randomly sampling a set of points in a given environment determined by a single categorical variable. A similar approach has been used formerly [52]. We extracted 2000 random coordinates from the “Closed to open shrubland” category of the North American Globcover map (Globcover 2009, http://due.esrin.esa.int/globcover [56]) to generate an unbiased dataset. The geographical extent of the virtual species was chosen to match the scale of the two real species ranges which are also both located in North America. In practice, the virtual species covered the larger part of western North America, including the lower valleys of the Rocky Mountains, Mojave Desert, Baja California peninsula, and Northern Mexico (Figure 2).

We additionally used the occurrence datasets of two real species (Figure 2). We compiled a set of 1825 occurrences for the painted turtle (*Chrysemys picta*) downloaded from the World Turtle Database (http://emys.geo.orst.edu; accessed on May 2011). The second species was the white-spotted slimy salamander (*Plethodon cylindraceus*) for which we collected a set of 208 observations. A part of the dataset was provided by J. Milanovich and additional records were obtained from the Global Biodiversity Information Facility (http://www.gbif.org, accessed on May 2011). According to our knowledge of the distribution of these species, these original datasets seem relatively unbiased, *i.e.* the distribution of records over space reflects the known spatial distribution of the species.

### Environmental predictors

We used distinct sets of environmental predictors depending on the modeled species. Climatic and topographic grids were downloaded from the WorldClim database [32] (http://www.worldclim.org) at a resolution of 2.5 arc-min (4.63 km at the equator). The global map of land cover provided by the European Spatial Agency was downloaded in its 2009 version (Globcover 2009 [56], http://due.esrin.esa.int/globcover) and rescaled to fit the 2.5 arc-min resolution of the other variables. Finally, we compiled 5 years of 10-day periods of NDVI (2007–2011), a measure of vegetation productivity derived from multispectral remote-sensing images, downloaded from the SPOT-VEGETATION project [57] (http://free.vgt.vito.be). We averaged across these 5 years three layers of mean, minimum and maximum annual NDVI.

We removed for each species some highly intercorrelated (correlation coefficient computed by ArcGIS 10; >0.9 or <−0.9) variables because multicollinearity may violate statistical assumptions and may alter model predictions [58]. The resulting variable sets were composed of 14 predictors (Table 1). Since the geographical distribution of the virtual species and *Chrysemys picta* records covered a large range in North America, we modeled both species across the same geographic area across North America. *Plethodon cylindraceus* occurrences are restricted to a smaller area of Eastern USA. Accordingly, the geographical range of predictors was restricted to a narrower area (Table 1).

**Table 1:** Environmental predictors included in MAXENT modeling for the virtual species, *Chrysmeys picta* and *Plethodon cylindraceus*.

	Virtual species	*Chrysemys picta*	*Plethodon cylindraceus*
**Variable**			
Altitude	×	×	×
Annual mean temperature	×	×	×
Mean diurnal range	×	×	×
Isothermality	×	×	×
Temperature annual range	×	×	×
Mean temperature of wettest quarter	×	×	×
Annual precipitation	×	×	×
Precipitation seasonality	×	×	×
Precipitation of warmest quarter	×	×	×
Precipitation of coldest quarter	×	×	×
Land cover	×	×	×
Maximum NDVI	×	×	
Mean NDVI	×	×	×
Minimum NDVI	×	×	×
Mean temperature of driest quarter			×
**Extent**			
Longitude			
min	−140.00	−140.00	−88.63
max	−50.00	−50.00	−70.68
Latitude			
min	20.00	20.00	28.91
max	75.00	75.00	45.46

The selected layers are indicated for each species, as well as the extent of modeling (in decimal degrees).

### Generation of sampling bias

**Figure 3:**
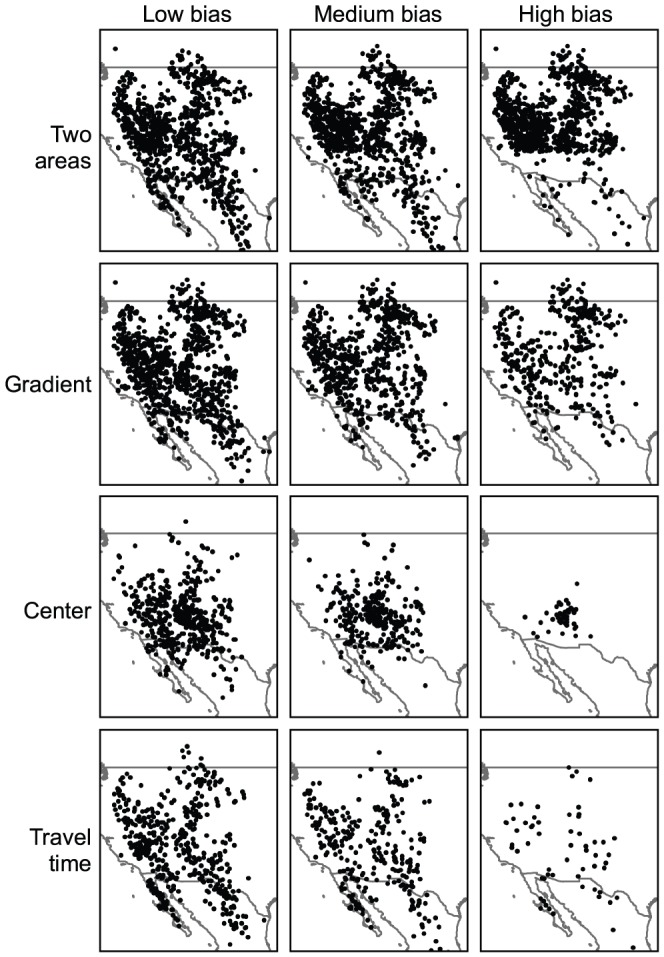
Generation of sampling bias for the virtual species. To generate artificial sampling bias, the original dataset (here the virtual species) was altered into 4 different types of bias (rows), each with 3 intensities (columns).

The three original datasets were altered to generate four types of bias that might occur when collecting observations (Figure 3). The original datasets were thus subsampled so that the remaining records were biased in the geographical space. We also created three levels of bias intensity, hereafter referred as “low”, “medium” and “high” to assess the effect of this parameter on model outputs (Figure 3). For each species, each combination of bias type (4) and bias intensity (3) was replicated 10 times resulting in a total of 360 biased datasets used to model distribution. The four types of sampling bias were generated as follows:

(1) TWO AREAS - The original dataset was biased such that its northern part exhibited a high density of records and the southern part a low density. This kind of bias is common when a species is systematically monitored in one part of its range and not surveyed in the other, for instance in different countries or groups of countries [44].

(2) GRADIENT - We generated a density gradient of observations decreasing from the north to the south of the range. This bias is close to the first one but here record density changed gradually. Such a bias would not reflect a difference in survey schemes between administrative divisions but a gradual reduction of sampling intensity towards a limit of species range.

(3) CENTER - The density of occurrences gradually decreased from the core of the distribution to the periphery. Such bias mimics cases in which sampling effort is concentrated in the centre of the known range of the species, whereas peripheral areas, potentially less suitable [59], are neglected.

(4) TRAVEL TIME - We used the travel time to the nearest city, using a map produced by the European Commission [60] (available at bioval.jrc.ec.europa.eu/products/gam). This variable integrates both the distance to the city and the presence of road networks. This map was used as a grid of sampling probability, in which probability of keeping a record was highest close to cities and in areas with dense road networks. This bias corresponds to a common situation where most of records are located around cities or along roads [35], [61].

The full details of the generation of sampling bias are given in Supporting information, Material S1.

### Species distribution modeling

We used for modeling the software MAXENT [30], a machine learning algorithm that applies the principle of maximum entropy to predict the potential distribution of species from presence-only data and environmental variables [26]. Currently, this widely used method is particularly efficient to handle complex interactions between response and predictor variables [15], [28], and is little sensitive to small sample sizes [29]. All models were computed using the version 3.3.3k of MAXENT (http://www.cs.princeton.edu/~schapire/maxent/). Runs were conducted with the default variable responses settings, and a logistic output format which results in a map of habitat suitability of the species ranging from 0 to 1 per grid cell, wherein the average observation should be close to 0.5 [15]. The models were evaluated by the area under the ROC curve (AUC), and three measures of overlap with the unbiased model (see below section “Model evaluation and statistical analyses”).

### Methods of sampling bias correction

We applied on all our biased datasets five methods of bias correction that have been already published. In order to evaluate their usefulness in real conditions, we used these methods as if the source, shape or strength of the sampling bias was unknown. Therefore, we did not select a correction method according to our knowledge of the bias, as this information is unknown in most empirical studies.

#### (1) Systematic Sampling

A subsample of records regularly distributed in the geographical space was selected [46], [54], [55], [62]. MAXENT already discards redundant records that occur in a single cell. We removed neighboring occurrences at a coarser resolution than MAXENT does. We created a grid of a defined cell size and randomly sampled one occurrence per grid cell. This subsampling reduces the spatial aggregation of records but does not correct the lack of data due to low sampling effort in some areas. This method could also underestimate the contribution of suitable areas where the high density or records reflects the true ecological value for the species. The resolution of the reference grid was 2 degrees for *Chrysemys picta* and the virtual species, and 0.2 degree for *Plethodon cylindraceus*.

#### (2) Bias File

This option is implemented in MAXENT. The software can be fed with a bias grid [49], [63] that is a sampling probability surface. The cell values reflect sampling effort and give a weight to random background data used for modeling. An ideal way of creating biasfiles would be to represent the actual sampling intensity across the study area. Although it can be roughly estimated by the aggregation of occurrences from closely related species [48], in most real modeling situations, this information is lacking. Thus, instead of using our knowledge of the artificially created biases, we produced bias grids by deriving a Gaussian kernel density map of the occurrence locations, rescaled from 1 to 20, following Elith et al. [63]. These maps were implemented in the biasfile option in MAXENT.

#### (3) Restricted Background

MAXENT, as most other presence-pseudoabsence methods, generates a “background” or “pseudo-absence” sample of points [15]. It has been argued that the selection of background points may strongly affect the resulting model [64]–[66]. By default 10000 pseudo-absences are randomly selected from the whole rectangular study area. This approach was followed for all the other cases, as most SDM studies keep the default MAXENT selection of background points. However, according to Phillips [47], if occurrences are restricted to a fraction of the study area, model performance can be enhanced by drawing the background points from this fraction of the area. The reliability of predictions should be improved when the model is transferred to the rest of the area. Following this recommendation, we randomly sampled 10000 pseudo-absences in buffer areas around occurrences and used them as background samples in MAXENT. Buffer size was a radius of 500 km for the virtual species and *Chrysemys picta*, and 100 km for *Plethodon cylindraceus*.

#### (4) Cluster

Biased datasets typically lead to spatial autocorrelation of records and artificial spatial clusters of observations thus violating the assumption of independence [67]. This bias can be circumvented by sampling one point per cluster in environmental space [53], [68], [69]. We first performed a principal component analysis (PCA) on the environmental descriptors of occurrences using the “ade4” R package [70] in order to define independent axes in the environmental space. Then, we ran a cluster analysis based on Euclidean distance on PCA axes space. The resulting dendrogram was used to define a number of classes corresponding to half of the occurrences. One record was randomly sampled per class and the models were run on these subsampled datasets.

#### (5) Split

When occurrence frequency greatly differs between two areas because of unequal sampling effort, the area can be split in two strata within which coverage probability is more homogeneous, and one model be computed for each stratum. This method has been used for species occurring over a large distribution range and extended environmental gradients [44], [51], [71]. We split our biased datasets in a northern and a southern stratum. We combined the model outputs to produce a composite model for the entire range, keeping the highest value in pixels where strata overlapped.

### Evaluation of correction methods

To estimate the ability of each correction method to recover the information contained in the original unbiased data set, we used 4 criteria that correspond to the interests of different end users of SDMs. We compared the models obtained after applying a bias in the dataset to the original model, and after applying a sampling bias correction method. The original dataset of the virtual species was created to be unbiased. The model computed from it will be thus referred as the unbiased model. Although we do not formally know the actual bias in the *Plethodon* and *Chrysemys* original datasets, we will also refer to the models computed from their original datasets as unbiased models. We should keep in mind that we compare the change in the resulting model, whatever the original bias. The models referred as biased were computed after applying a sampling bias and the corrected models after applying a correction method to the biased dataset.

#### (1) AUC

The area under the receiver operating curve (ROC), known as the AUC is one of the most common statistics to assess model performance. AUC can be interpreted as the probability that a presence cell have a higher predicted value than a absence cell (or pseudo-absence), both of them being chosen randomly [28]. Although the use of AUC for the evaluation of ecological models has been criticized, especially when calculating against background points rather than true-absences [72], [73], it should be reliable to compare models generated for a single species in the same area and the same predictors.

The calculation of AUC was performed using the R package “PresenceAbsence” [74], using as test points the fraction of the original dataset which was excluded to create the biased dataset. The metrics was calculated by the comparison between these test points and either (i) 10000 true absence points sampled outside the range, which corresponds here to the true occupancy for the virtual species, or (ii) 10000 background points randomly sampled in all the study area for the real species. For original models, in the absence of true test points, a mean AUC value was computed using 5 random splits of the dataset, each subsample being used in turn to evaluate the model.

#### (2) *D_GEO_* overlap in the geographical space

Several metrics of niche overlap are available (e.g. *D* [75], modified Hellinger distance *I* [76] or *BC* [77]). We used the Schoener's *D* index [75] that has been suggested to be best suited for SDM outputs [78]. This statistics considers the probability distributions across space of the difference in the probability of presence of two species, based on their respective distribution models. *D_geo_* index is ranged from 0 (no overlap) to 1 (complete overlap, identical models).

#### (3) *D_ENV_* overlap in the environmental space

We estimated niche overlap in the environmental space between the unbiased, biased, and corrected models. We used the PCA-env approach described by Broennimann [79] to calculate Schoener's *D* index based on the environmental characteristics of two sets of occurrences. This approach defines the environmental space by the two first axes of a principal component analysis of all the pixels of the study area. The niche overlap is calculated from the smoothed density of occurrences in the environmental space following a kernel density function applied on each dataset. Depending on the correction method used, some models can be based on the same input dataset (*e.g.* the biasfile correction uses the same records as the biased model). In order to compare our SDMs, we applied the PCA-env method on 500 points sampled from the SDM outputs instead of on the input records. We selected these points in the output map using the SDM probability of presence as a sampling probability.

#### (4) *G_OVER_* overlap between binary maps

SDM outputs are often converted to binary maps that are more tractable for conservationists who for instance need to delineate protected areas. In such maps, a pixel is considered as either suitable to the species or not. We used the non-fixed 10^th^ percentile training presence threshold value to generate binary maps as proposed by Liu et al. [80]. We then measured the overlap between biased and corrected models, and unbiased models. This measure strictly measures the geographical overlap whereas the *D* index estimates the overlap of ecological niches in environmental space (*D_env_*) or projected onto the geographical space (*D_geo_*).

To evaluate the performance of bias correction, we derived new indicators from AUC, *D_geo_*, *D_env_*, and *G_over_* that quantify the improvement of the corrected model to the biased model, standardized by the difference between the unbiased and the biased models. These indices, named respectively ΔAUC, Δ*D_geo_*, Δ*G_over_* and Δ*D_env_* were calculated as follows:
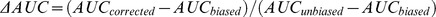


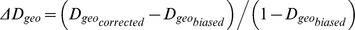


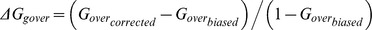


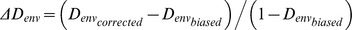



These four indices range from -∞ to 1, a positive value indicating that the model was actually corrected (with 1 corresponding to perfect correction, *i.e.* corrected model exactly similar as the unbiased one) whereas a negative value indicates that the correction produced a worse model than the biased one.

## Results

### Effect of bias type and intensity on model outputs

**Figure 4:**
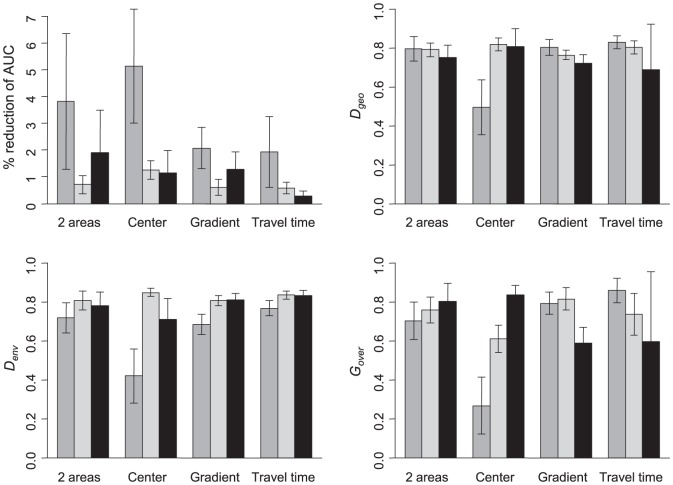
Evaluation indices of biased models across bias intensities. Reduction of AUC between unbiased and biased models (in percentage), Schoener's *D* overlaps between biased and unbiased models computed on SDMs (*D_geo_*) and in environmental space (*D_env_*), and *G_over_* the binary distribution overlap (mean ± SD). Dark grey bars: *Chrysemys picta*, light grey bars: *Plethodon cylindraceus*, black bars: virtual species.

The biased models resulted in a reduction of AUC in all cases and a deviation from the unbiased model for all overlap measures (Figure 4). The deviation varied largely depending on species and bias type though: 0.28–0.91 for *D*_geo_, 0.18–0.89 for *D_env_*, 0.03–1 for and *G_over_*. For the virtual species, the bias type yielding the largest deviation depended on the performance measures considered: “2 areas” for the AUC, “travel time” for *D_geo_*, “Center” for *D_env_* and “Gradient” for *G_over_*, the latter providing the lowest values of overlap with the original unbiased models. However, the differences between unbiased and biased models remained moderate (mean percentage variation: AUC: −1.74%; *D_geo_*: −24.41%; *D_env_*: −24.62%; *G_over_*: −30.07%). All bias types had also globally similar effects in terms of deviation from the unbiased model (Figure 4). For *Chrysemys picta*, all evaluation measures were strongly affected by the “center” bias. AUC decreased more than 5%, and overlaps with the unbiased model ranged from 0.26 to 0.49. In contrast, the *P. cylindraceus* dataset was overall weakly affected by the biases so that the biased models did not lead to noticeable differences with the unbiased model. The strong effect of the “center” bias was visible in *Plethodon cylindraceus* only for the overlap of binary maps (*G_over_*). This bias in which only the central zone is sampled may exclude a large part of the original environmental space and lead to very inaccurate SDM outputs. Interestingly, the decrease in AUC performance for all bias types was more pronounced in *C. picta* than the two other datasets even when the values of overlap were in the same range.

### Relative performance of correction method

Since we evaluated the performance of correction methods using indices with different sets of assumptions, interpretation may slightly differ with the measure considered. However, as we mainly aimed at comparing SDM outputs, *i.e.* maps of habitat suitability, we primarily focused our interpretations on Δ*D_geo_* which truly evaluates the overlap between standard SDM maps. Moreover, the two measures based on Schoener's *D*, in geographic (Δ*D_geo_*) and in environmental space (Δ*D_env_*), were highly correlated and outputs of bias correction were qualitatively similar (Supporting information, Figure S1). We discuss here results for Δ*D_geo_* only.

Correction performance strongly depended on the species (Table 2). Considering the three species together, less than half (29%) of all combinations (species × bias type × bias intensity × correction method) yielded corrected models (following Δ*D_geo_*) with more accurate predictions than the biased model. For the virtual species, and considering Δ*D_geo_*, 57% of corrected models (34 out of 60 combinations of bias type, bias intensity and correction method) were more similar to the model generated with the unbiased dataset than the biased model (Table 2). Most cases for which no method was able to provide bias correction were “center” and “travel time” biases, with medium to high intensities. Conversely, only 7% of *P. cylindraceus* models were corrected (4 cases out of 60), while 25% of *C. picta* models were corrected (15 cases out of 60) and offered a better result than the biased model.

**Table 2:** Mean correction performance across 10 replicates, for each species, bias type, bias intensity and correction method.

	*2 areas*			*Center*			*Gradient*			*Travel time*		
	low	medium	high	low	medium	high	low	medium	high	low	medium	high
**(a) Δ** ***AUC***												
***Chrysemys picta***												
Biasfile	−0.64	−0.1	−0.01	−0.07	−0.03	0.25*	−0.93	−0.61	−0.31	−2.09	−0.57	−0.2
Cluster	**0.09**	**0.09**	**0.28**	**0.24**	**0.19**	**0.12**	−0.08	−0.06	**0.01**	−1.12	−0.22*	−0.05*
Restricted background	−0.02	−0.21	−0.16	−0.43	−0.69	−0.85	−0.12	−0.22	−0.16	−0.96	−0.46	−0.62
Split	0.60*	0.64*	0.74*	−0.12	−0.14	**0.1**	0.10*	0.02*	0.14*	−0.94*	−0.28	−0.43
Systematic sampling	−0.85	**0.19**	**0.42**	0.31*	0.33*	−0.82	−0.97	−0.58	−0.16	−3.76	−1.17	−0.26
***Plethodon cylindraceus***												
Biasfile	**0.12**	**0.14**	−0.03	**0.1**	**0.09**	**0.22**	**0.03**	−0.24	−0.11	−0.07	−0.02	**0.02**
Cluster	**0.17**	**0.11**	**0.15**	**0.19**	**0.19**	**0.26**	**0.03**	0.00*	−0.12	**0.02**	0.02*	−0.08
Restricted background	−10.03	−10.45	−10.49	−5.36	−4.12	−5.04	−6.19	−10.64	−7.88	−12.74	−9.47	−11.12
Split	**0.03**	**0.01**	**0.2**	−0.12	−0.04	**0.2**	−0.13	−0.09	−0.18	−0.07	−0.07	−0.24
Systematic sampling	0.31*	0.32*	0.42*	0.51*	0.51*	0.50*	0.13*	−0.09	0.14*	0.17*	**0.01**	0.04*
**Virtual species**												
Biasfile	−3.3	**0.42**	**0.49**	**0.17**	0.35*	**0.26**	−0.1	**0.34**	**0.41**	−2.87	−2.40*	−0.27*
Cluster	−0.75	**0.11**	**0.24**	−0.18	−0.15	−0.17	−0.01	**0.09**	**0.07**	−1.82	−3.3	−0.43
Restricted background	−2.09	−0.34	−0.78	−1.97	−1	**0.05**	−1.04	−1.26	−1.5	−9.87	−18.76	−6.43
Split	0.18*	0.72*	0.82*	−0.2	−0.29	−0.17	0.38*	0.42*	0.60*	−1.80*	−2.48	−0.53
Systematic sampling	−3.26	**0.45**	**0.72**	0.39*	**0.33**	0.35*	−0.83	−0.08	**0.39**	−2.54	−2.77	−0.69
**(b) Δ** ***D_geo_***												
***Chrysemys picta***												
Biasfile	−0.41	−0.21	**0.03**	**0.3**	**0.28**	**0.31**	−0.49	−0.32	−0.13	−0.72	−0.56	−0.28
Cluster	−0.34	−0.28	−0.13	**0.01**	**0.08**	**0.03**	−0.27	−0.13*	−0.03	−0.18*	−0.14*	0.00*
Restricted background	−0.31	−0.19	−0.11	−0.11	−0.09	−0.12	−0.19*	−0.16	−0.09	−0.28	−0.33	−0.44
Split	−0.24*	0.07*	0.37*	−0.14	−0.08	**0.04**	−0.27	−0.23	−0.05	−0.37	−0.28	−0.21
Systematic sampling	−0.47	−0.19	**0.02**	0.41*	0.39*	0.32*	−0.47	−0.25	0.02*	−0.59	−0.31	−0.07
***Plethodon cylindraceus***												
Biasfile	−1.17	−1.14	−0.88	−1.1	−0.75	−0.62	−0.96	−0.98	−0.94	−1.2	−0.76	−0.61
Cluster	−0.05*	−0.07*	−0.02*	−0.17	−0.09	−0.03	−0.15	−0.12*	−0.14	−0.01*	−0.06	**0.02**
Restricted background	−3.95	−2.72	−2.27	−4.55	−3.64	−3.08	−2.15	−2.3	−1.95	−3.8	−3.08	−2.54
Split	−0.12	−0.23	−0.73	−0.16	−0.05	−0.02	−0.14	−0.23	−0.26	−0.18	−0.09*	−0.07
Systematic sampling	−0.16	−0.29	−0.19	−0.06*	0.04*	0.01*	−0.10*	−0.19	−0.09*	−0.24	−0.1	0.03*
**Virtual species**												
Biasfile	**0.14**	**0.46**	**0.09**	0.61*	−0.64*	−1.35	**0.36**	**0.25**	**0.47**	0.79*	−0.09*	−0.07*
Cluster	**0.28**	**0.32**	−0.55	**0.2**	−2.1	−2.44	**0.41**	**0.18**	**0.31**	**0.75**	−0.44	−0.27
Restricted background	0	**0.2**	−0.76	−0.07	−2.74	−2.89	**0.25**	−0.22	-0.14	**0.53**	−1.76	−1.4
Split	**0.43**	0.58*	0.32*	**0.1**	−2.51	−2.35	0.49*	**0.25**	**0.41**	**0.72**	−0.64	−0.51
Systematic sampling	0.32*	**0.51**	**0.14**	**0.41**	−1.35	−0.99*	**0.45**	0.30*	0.50*	**0.78**	−0.14	−0.42
**(c) Δ** ***G_over_***												
***Chrysemys picta***												
Biasfile	**0.18**	**0.25**	**0.09**	**0.26**	**0.07**	**0**	−0.10*	−0.05	−0.23	−0.11*	−0.19	−0.2
Cluster	−0.37	−0.14	−0.07	−0.01	**0.06**	**0.01**	−0.38	−0.09	−0.15	−0.55	−0.45	−0.84
Restricted background	−0.12	−0.09	−0.05	**0.01**	0	−0.01	−0.14	−0.13	−0.08	−0.2	−0.32	−0.10*
Split	−0.01	**0.08**	0.57*	−0.04	−0.05	0	−0.53	−0.56	−0.18	−0.99	−0.9	−1.25
Systematic sampling	0.33*	0.40*	**0.31**	0.65*	0.41*	0.34*	−0.25	0.05*	0.18*	−0.23	0.25*	−0.98
***Plethodon cylindraceus***												
Biasfile	**0.29**	0.74*	**0.52**	0.71*	0.72*	0.63*	0.48*	**0.33**	0.43*	0.43*	0.29*	0.32*
Cluster	**0.29**	**0.3**	**0.23**	**0.39**	**0.14**	**0.11**	**0.08**	**0.39**	**0.15**	**0.26**	−0.23	**0.11**
Restricted background	−0.62	−0.19	−0.45	−0.34	−0.17	−0.21	**0**	−0.35	−0.17	−1.09	−0.79	−0.87
Split	0.52*	**0.4**	0.83*	**0.54**	**0.51**	**0.32**	**0.41**	0.48*	**0.29**	**0.19**	−0.09	−0.03
Systematic sampling	**0.48**	**0.65**	**0.62**	**0.67**	**0.49**	**0.46**	**0.26**	**0.12**	**0.21**	0.43*	**0.01**	**0.31**
**Virtual Species**												
Biasfile	0.41*	0.77*	−0.31	−0.25	−2.33	−3.09	0.80*	0.65*	**0.66**	0.86*	−0.39	−0.44
Cluster	−0.21	**0.2**	−2.37	−1.13	−3.53	−3.05	**0.61**	**0.29**	**0.4**	**0.8**	−0.73	−0.62
Restricted background	0	**0.34**	−2.21	−1.17	−3.7	−3.17	**0.69**	**0.39**	**0.41**	**0.83**	−0.53	−0.42*
Split	**0.31**	**0.62**	0.02*	−1.16	−3.68	−3.03	**0.69**	**0.47**	**0.61**	**0.83**	−0.64	−0.57
Systematic sampling	**0.05**	**0.62**	−0.07	0.02*	−1.37*	−2.01*	**0.62**	**0.42**	0.73*	0.86*	0.17*	−0.72

Positive values, *i.e.* cases where the bias was actually corrected, are shown in bold. For each combination of species and bias (type × intensity), the best method (*i.e.* the one which has the highest value) is highlighted by an asterisk. Correction performance is estimated by three measures: (a) Δ*AUC*, (b) Δ*D_geo_*, (c) Δ*G_over_*.

**Figure 5:**
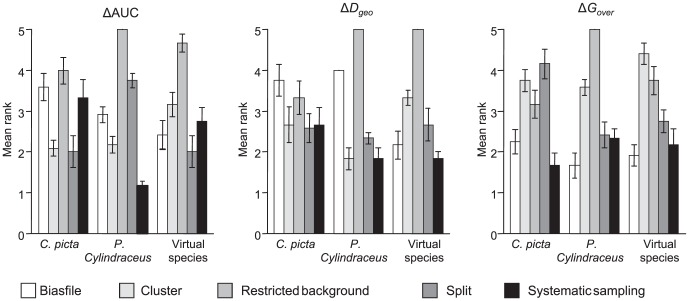
Rank of each method to correct sampling bias. Mean ranks ± standard-error for the performance of each method to correct sampling bias for each species (*Chrysemys picta*: left, *Plethodon cylindraceus*: center, *virtual species*: right), following 3 measures of correction performance: ΔAUC (left), Δ*D_geo_* (centre), and Δ*G_over_* (right). For each type of bias and bias intensity, the method which results in the most efficient correction is set to 1 whereas the least powerful method is set to 5. The plotted values are the mean rank across the 4 types of bias and 3 intensities.

Regardless of the species, the bias type, and the metrics considered, the restricted background method failed to improve the biased models in almost all tested cases. The other methods performed better but were ranked differently depending on bias type. Systematic sampling performed slightly better and more consistently among the competing methods as shown by the relative performance of each method across bias types (Figure 5). Although systematic sampling was not always ranked first, it showed very little deviation from the most performing method and performed on average better than the others (for Δ*D_geo_*: mean rank _Systematic sampling_  = 2.11±1.08 SD; mean rank _Split_  = 2.53±1.08 SD, mean rank _Cluster_  = 2.613±1.23 SD; mean rank _Biasfile_  = 3.31±1. 31 SD). In contrast, the restricted background method recorded the least correction (mean rank _Restricted background_  = 4.44±1.13 SD).

Overall, and considering only Δ*D_geo_*, the systematic sampling method was able to correct the bias (Δ*D_geo_*>0) in 33% of the test cases. This success rate rose to 66% in the case of the virtual species, for which we were able to compare to a true unbiased model. However, the biasfile corrected the initial bias in 23% of test cases. The cluster and split method were both efficient in 23% of cases while only 6% of cases were corrected by the restricted background method.

Interestingly, relative performance between methods was consistent across metrics (Figure 5). The restricted background method was always the least performing one in terms of ΔAUC, Δ*D_geo_* and Δ*G_over_* (but for *C. picta* and Δ*D_geo_*, for which it is ranked 4/5). The systematic sampling method was among the most performing methods. However, even if systematic sampling was overall the most efficient method across species in terms of Δ*D_geo_*, it was outperformed by the split or biasfile methods in some cases for the virtual species, or by the cluster method under some combinations of bias × intensity for the two real species (Table 2). However, when the systematic sampling was unable to resolve the bias, this latter was most often equally poorly corrected by any of the methods tested.

## Discussion

As an unexpected first finding, we noticed that the range of AUC values obtained for biased and corrected models remained high even for models with the strongest biases. The decrease in AUC observed after applying the bias was moderate, less than 2% on average, across species and bias type. Moreover, the AUC values of the biased models were almost always over 0.8 or 0.9, which would classify the models as “good” or “very good” (Araújo et al. [81] adapted from Swets [82]). Together with other studies [72], [73], [83] our results highlight that this measure may poorly reflect model accuracy. Therefore, studies that focus solely on the AUC value should interpret their results with caution. AUC may be a good statistical measure of discrimination ability, but it often fails to quantify the ecological realism of modeled distribution [72], [73], [83] especially when estimated from presence-only data. Because we have a reference model, we will mainly focus on the overlap indices with the unbiased model as a measure of predictive accuracy performance.

Contrary to previous studies investigating sampling bias correction in SDM that focused on a few methods and simple biases [41], [52]–[54], we reviewed here five different ways to deal with sampling bias and used both real and virtual datasets under various bias scenarios. We also considered bias intensity that has been to our knowledge never assessed and proved to be of as a high concern as the type of bias. Moreover, instead of relying only on classical measures of SDM performance such as AUC (as used in Syfert et al. [41], Varela et al. [53] and Boria et al; [54]) or omission/commission error (as used in Kramer-Schadt et al. [52]), we evaluated the correction performance by directly comparing the SDM outputs. Therefore, we actually assessed the ability of the tested methods to recover the unbiased model, which is the expected behavior of an efficient sampling bias correction. In addition, rather than basing our conclusions on island species [41], [52], [54], we used continental species whose distributions are clearly shaped by climate and not by a geographically bound space.

Our results clearly evidence that the different methods of sampling bias correction tested here may have very variable efficiency depending on the modeling conditions (biases type and correction method). Interestingly, the correction may have a positive effect, and actually contributes to correct the bias; nonetheless, in some cases it may produce a poorest model than the biased model. These results suggest that the problem of sampling bias in species distribution modeling has probably multiple answers depending on the context. We especially emphasize that the type and intensity of bias influence the ability of various methods to resolve the initial bias.

However, correction methods did not perform equally across the various conditions. The less efficient method restricted the spatial extent of the background whereas in other methods, the background points were selected from the whole available environment (*i.e.* randomly drawn from the area covered by the environmental grid files). Surprisingly, this method is often used and have been contributed to improve SDM performance in some cases [47], [48]. However, as suggested by Thuiller et al. [84] and Vanderwal et al. [85], excessively restricting the geographical extent of pseudo-absences to a narrow area or selecting them from a too large area reduces model accuracy. Background selection may greatly influence the resulting model as it determines the underlying assumptions of the model to use [64]. Therefore, this step should be undertaken with caution. The size of the buffer used for background selection also greatly influences model performance. For instance, AUC often increases with the size of the study area because it contributes to include background points that have environmental characteristics greatly distant from the species requirement, resulting in artificial increase of SDM validation [65]. The selection of the training area should therefore be strictly relevant to the ecology of the species and the objective of the study. A relevant selection of the training area (the geographic region in which background points are selected) should reflect the geographical space accessible to the species over a given time period [65]. It may thus be essential to carry out a rigorous investigation of the optimal geographic distance between the set of occurrences used to train the model and background points. It has to be both optimal for model training and biologically meaningful. The interpretation of the modeled distribution must also be engaged carefully as it may reflect the fundamental niche or the true occupied range, and often a position between both.

Regarding the high variability in correction performance of the different methods depending on various factors, it is difficult to propose a universal guideline to solving sampling bias. It might be advisable to evaluate first several types of correction. The final choice of correction method would be then based on their effect in classical SDM evaluation metrics (*e.g.* AUC, Kappa, True Skill Statistics TSS) and possibly the adequacy of output maps to *a priori* knowledge of the species distribution. A first useful step might also be to evaluate bias type to design and select the most appropriate correction. For instance, the split method makes sense only if the most and less sampled areas are at least roughly known. Most of the time, sampling bias is only inferred by the known sampling effort or empirical knowledge of the species distribution. The true severity and shape of this bias is almost always lacking. However, in some cases, bias can be evaluated by comparing the geographic distribution of the available occurrences to known sources of bias. An estimation of sampling probabilities across the study area can provide insights on the potential bias that may affect the collected observations and help in further choice of the correction method. The known characteristics of the modeled species may also condition the strategy to use. A species with an expected very large geographical and/or environmental range should benefit from being split into two or more partitions that are combined afterward [51]. Finally, the five methods tested here are not necessarily exclusive. For instance, it is possible to split a large dataset and apply systematic sampling to each dataset in species with broad distributions [44] or to apply the biasfile method after using first another correction method

Nevertheless, we found that only systematic sampling constantly performed well irrespective of species and bias type. Interestingly enough, it happened to be the simplest and most obvious way to solve the geographic bias. Beside, this method can be quickly applied to any dataset, even if the nature of the bias is unknown. Kramer-Schadt et al. [52] and Boria et al. [54] also identified this technique, which they named spatial filtering, as the most effective (but note that Varela et al. [53] found that environmental filtering, equivalent to our cluster method, may provide better results). We might keep in mind that this method may have a few drawbacks though. Subsampling the complete dataset may alter the distribution of occurrences in the environmental space and exclude some portion of environmental space from the input records. This problem may be circumvented by adjusting the grid size but this may not be possible when the sample size of occurrences (presences) is too low. On the contrary, smoothing the distribution of occurrences may lead to overestimating the probability presence in marginal areas. The grid cell size used to sample the occurrences may also be a source of problem; it must be large enough to resolve the bias but not too large to result in a strong loss in resolution. A fine adjustment of the parameters of the method (*e.g.* the resolution of systematic sampling that provides the optimal trade-off between sampling bias correction and information reduction) is thus necessary to maximize its performance.

Our results suggest that the difference in performance between the systematic sampling method and the optimal method for the dataset under investigation is slight. Since systematic sampling seems to be robust enough to differentiate sampling bias and species, we suggest that this could be the method selected first if no further attempts to correct sampling bias are to be made. This method provides a quick and efficient way to improve SDM from a biased dataset and is likely to be relevant most of the time. Even if the elaboration of a step-by-step framework would be ideal to assist in the definition of a reliable strategy of bias correction, we highlight that most SDMs studies, at least those that use MAXENT as modeling algorithm, would highly benefit from this simple tweaking of input occurrences. Regarding the major impacts that conservation actions are expected to provide [86], [87], they must be based as much as possible on irrefutable models. Therefore, it is essential to develop simple methods intended for conservation practitioners, such as techniques of sampling bias correction, to build robust distribution models. In this regard, we encourage MAXENT users to carefully take sampling bias into account, and to use systematic sampling of their input occurrences as a quick and simple resolution of bias.

## Supporting Information

Figure S1 **Correlations between measures of correction performance.**
(PDF)

Material S1 **Details of the generation of sampling bias.**
(PDF)
