# Supplementary material for: Mapping Species Distributions with MAXENT Using a Geographically Biased Sample of Presence Data: A Performance Assessment of Methods for Correcting Sampling Bias
Source: PLoS One. 2014 May 12;9(5):e97122. doi: 10.1371/journal.pone.0097122 (PMC4018261; doi:10.1371/journal.pone.0097122)

**Figure S1:** Correlations between measures of correction performance. Lower panel: correlation scatterplots, upper panel: Pearson's correlation coefficient *r* and associated p-value.

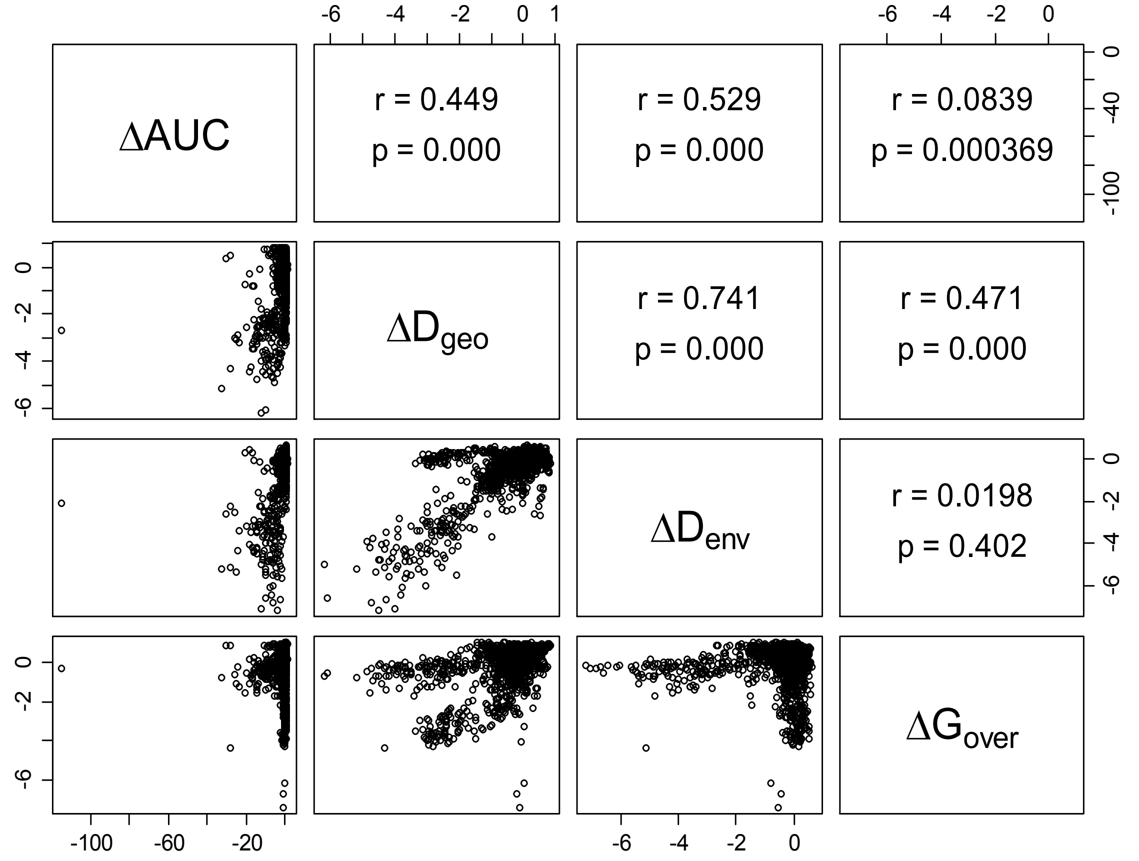

Supplement: Figure S1 — Correlations between measures of correction performance. (PDF) [file pone.0097122.s001.pdf]
