# Supplementary material for: Mapping Species Distributions with MAXENT Using a Geographically Biased Sample of Presence Data: A Performance Assessment of Methods for Correcting Sampling Bias
Source: PLoS One. 2014 May 12;9(5):e97122. doi: 10.1371/journal.pone.0097122 (PMC4018261; doi:10.1371/journal.pone.0097122)
Supplement: Material S1 — Details of the generation of sampling bias. (PDF) [file pone.0097122.s002.pdf]

Supplementary material S1: Details of the generation of sampling bias.

(1) Two areas – The original set of species occurrences was divided into a northern set and a southern set following the median latitude of available records. The northern set was defined as the area of high density of records whereas the southern set was defined as the area of low density of records. In order to create the biased density, we randomly removed occurrences in each subset, keeping a higher proportion of records in the northern set. For the high bias, 95% of records were kept in the north and 5% in the south. For the medium and low biases, the ratio of observation was respectively 80% / 20% and 70% / 30%. The two datasets were thereafter merged to be used in modeling. As a result, the number of occurrences was the same for each bias intensity, corresponding to the half of original records.

(2) Gradient – We generated a sampling probability function depending on latitude, ranging from 1 for the northernmost point to 0 for the southernmost point. We randomly selected occurrences from the original dataset following this probability function. The low bias was generated following a linear sampling probability function while the medium and high biases followed an exponential function. The intensity of bias was modulated according to the following equations, where *y* is the latitude:

Low intensity: $p = 1 - \left(\frac{\max(y) - 1}{\max(y) - \min(y)}\right)$ $p = 1 - \left(\frac{\max(y) - 1}{\max(y) - \min(y)}\right)$

Medium intensity: $p = 1 - \left(\frac{\max(e^{xy}) - e^{xy}}{\max(e^{xy}) - \min(e^{xy})}\right)$ $p = 1 - \left(\frac{\max(e^{xy}) - e^{xy}}{\max(e^{xy}) - \min(e^{xy})}\right)$ where $x = 0.1$

High intensity: $p = 1 - \left(\frac{\max(e^{xy}) - e^{xy}}{\max(e^{xy}) - \min(e^{xy})}\right)$ $p = 1 - \left(\frac{\max(e^{xy}) - e^{xy}}{\max(e^{xy}) - \min(e^{xy})}\right)$ where $x = 0.2$

(3) Center - The records were selected from sampling probability functions depending on the distance to the geographical centroid of occurrences. Here, the probability was highest at the centroid of the original set of points and decreased towards the periphery, according to the following equation, where *d* is the distance to centroid:

$$p = 1 - \left(\frac{\max(e^{xd}) - e^{xd}}{\max(e^{xd}) - \min(e^{xd})}\right)$$

$$p = 1 - \left(\frac{\max(e^{xd}) - e^{xd}}{\max(e^{xd}) - \min(e^{xd})}\right)$$

The intensity of bias was modulated by varying the value of *x*. Low bias: $x = 1/3$, medium bias: $x = 1/5$, high bias: $x = 1$

(4) Travel time - For each occurrence point, we extracted the corresponding travel time (extracted from the map produced by the European Commission (Nelson 2008) available at bioval.jrc.ec.europa.eu/products/gam/) and used this value as a sampling probability weight. The three bias intensities were generated by sampling respectively 25%, 15% and 5% of original records for the virtual species and *Chrysemys picta*. Because of the lower number of available records, we sampled 50%, 30% and 20% of original dataset for *Plethodon cylindraceus*.
